# Patients and relatives coping with inflammatory arthritis: Care teamwork

**DOI:** 10.1111/hex.12982

**Published:** 2019-11-27

**Authors:** Morgane Brignon, Christel Vioulac, Emilie Boujut, Caroline Delannoy, Catherine Beauvais, Joelle Kivits, Didier Poivret, Janine‐Sophie Giraudet Le Quintrec, Aurélie Untas, Anne‐Christine Rat

**Affiliations:** ^1^ EA 4360 APEMAC Université de Lorraine Nancy France; ^2^ Laboratory of Psychopathology and Health Processes EA 4057 University of Paris Descartes – Sorbonne Paris Cité Boulogne‐Billancourt France; ^3^ EMASP ‐ Soins de supports Foch Hospital Suresnes France; ^4^ Hôpitaux universitaires Est Parisien AP‐HP Rheumatology Department Saint‐Antoine Hospital Paris France; ^5^ Metz‐Thionville Hospital Metz France; ^6^ Centre d’Education Thérapeutique pour les Rhumatismes Chroniques (CERC) Cochin Hospital AP‐HP Paris France; ^7^ Rheumatology Department University Hospital Nancy Nancy France

**Keywords:** caregivers, inflammatory arthritis, relatives

## Abstract

**Objective:**

To explore how patients and relatives experience and talk together about their life with inflammatory arthritis.

**Design:**

Qualitative research.

**Setting:**

A convenience sample was used. Participants were recruited in seven rheumatology departments in France.

**Participants:**

Patients with rheumatoid arthritis or spondyloarthritis, agreeing to participate in the study with a relative, age at least 18 years.

**Data collection and analysis:**

Two psychologists conducted face‐to‐face interviews with 20 patient‐relative dyads (40 individuals). A thematic analysis followed a general inductive approach.

**Results:**

Saturation was reached after interviews with 20 dyads. The analysis revealed four main themes: (a) disease ‘lived’ together: a new role for the relative (providing help in physical tasks, emotional support, acting as a driving force, having a role in medical care) and communication around the disease (not focusing on the disease); (b) impact of the disease on the relationship; (c) social impact of the disease on the dyad (social isolation); (d) difficulties and needs of the relative (need to better know the disease).

**Conclusion:**

This study has highlighted the importance of recognizing the role of the relative in the management of inflammatory arthritis disease, especially when medical decisions are shared with professionals. A joint approach to treatment is a basis for coping with the disease. This approach supposes (a) discussions about relatives’ new roles to clarify them, (b) patients’ and relatives’ communication skills and (c) a good understanding of each other, which can be improved by providing information on the disease and coping strategies for both the patient and the relative.

## 1 BACKGROUND

The transition from a state of good health without disease to a state in which one must live on a daily basis with a lifelong disease often depends on practical and emotional support and care from relatives, especially partners.^1^

Symptoms of inflammatory arthritis (IA) (rheumatoid arthritis [RA] or spondyloarthritis [SpA]), especially pain or fatigue,^2^ are not always visible and are unpredictable, which has specific consequences, especially on relationships with others.^3^ In these diseases, help in physical tasks and emotional and social support are crucial,^4^, ^5^, ^6^, ^7^, ^8^ and adjustment to the disease necessarily 9 implies adjustment for those who live close to the person.^10^ Supportive relationships seem to foster better coping strategies to adjust to rheumatic diseases, which leads to better psychological adjustment.^9^

Relatives’ and patients’ adjustment to a disease is complex, and there is room for improvement in the support provided to the dyad. In rheumatology, the participation of relatives in therapeutic patient education (TPE) interventions is often limited to relatives participating in a few sessions with the patients and rarely relatives participating in a dedicated session 11 or active involvement in the intervention.^12^ In RA, results of TPE targeting relatives are rare and controversial.^8^, ^13^ EULAR recommendations for patient education for people with IA 14 point to the need ‘to develop and evaluate TPE for significant others (partners, spouses, family and carers)’.

The first step in developing interventions to support dyads is to study their needs, functioning and communication. To highlight the interactions within the dyad, dyad partners were interviewed together, which has rarely been done, especially in IA.^15^ They had the opportunity to express themes, difficulties, mistakes or how they cope together. Shared interviews have been used for couples with other long‐term diseases. For example, in couples with one individual having diabetes and osteoarthritis, couples described their coping activities as individual, shared or a mix of individual and shared efforts.^16^ For couples with one individual having multiple sclerosis, shared interviews highlighted how roles and responsibilities for the information search can change over time, often depending on symptoms. Usually, only one family member, the patient or their partner, takes this responsibility.^17^ For couples with one individual having cardiovascular disease, some assessed the illness as a transformative experience in their lives, bringing them closer together. Some couples experience the illness as a threat for both partners, and the disease is faced as a mutual task. Other couples assessed diverse possibilities for positive change but did not achieve them.^18^

The objective of this study was to explore patients’ and relatives’ experience of IA and their difficulties, relationship, communication, coping strategies and needs. The results may help develop TPE interventions involving relatives to support them in their difficulties and in assuming their role and to optimize communication and the relationship to improve the adjustment of the dyad to the disease.

## 2 METHODS

### 2.1 Sample

A convenience sample was used.^19^ Participants were recruited by rheumatologists during their consultations in seven rheumatology departments located in medium or large cities from different regions in France with a coverage of urban or rural areas.

Inclusion criteria for patients were RA or SpA, agreeing to participate in the study with a relative and age at least 18 years. Exclusion criteria were a major comorbidity that might increase the burden of IA (eg, severe heart failure, neurological disease). Relatives were invited to participate by patients or directly during a consultation when they were present. Relatives were defined as a close relative or a person who shows a special interest in the person.

### 2.2 Interviews

After having obtained written informed consent, patients and relatives were first invited to complete a self‐reported questionnaire regarding sociodemographic and medical status. Then, between October 2014 and July 2015, three women health psychologists (CV PhD in psychology; CD master's degree in psychology) conducted face‐to‐face interviews with the dyad (patient and relative interviewed together) in the hospital centre in which the patient was followed up.

Researchers were all trained to conduct interviews; they had experience in caring for patients with a long‐term disease in general but were not specialized in patients with IA care. They did not know any of the patients or relatives before the study commencement. Two were specialized in health psychology research, and one was a clinical psychologist.

Only the researcher and the dyad were present during the interviews. Interviews lasted 60 to 90 min. They were audio‐recorded and then transcribed verbatim. The interviews were semi‐structured and conducted until data saturation was achieved (no new concepts emerge during the last interviews).^20^, ^21^ None of the interviews were repeated, and transcripts were not returned to participants for comments. Field notes were not taken during or after the interviews.

The interview schedule (Table 1) was built from a literature review^22^ performed by the project working group consisting of rheumatologists with experience in TPE, a methodologist, psychologists and a patient from a patient association. The interview schedule was tested with 3 patients, but no change was needed.

**Table 1:** The interview schedule

How would you describe your relationship? ○ *Follow‐up on the connections with the disease* Do you talk about illness together? ○ How? ○ *Investigate the themes of the exchanges (practical and emotional aspects)* What is the impact of the disease on your relationship? ○ *In everyday tasks, emotional, social* Which difficulties do you face? ○ *Symptoms, physical, emotional, social* What help could health professionals give you about it?

### 2.3 Other data collected

Both patients and relatives provided data on sociodemographic characteristics (age, sex, education level, occupation) and self‐reported measures assessing disease activity (Routine Assessment of Patient Index Data [RAPID3]^23^ and Bath Ankylosing Spondylitis Disease Activity Index [BASDAI]), comorbidities (Groll functional comorbidity index), anxiety and depression (Hospital Anxiety Depression Scale [HADS]^24^) and experience of the caregiver (Caregiver Reaction Assessment [CRA],^25^ Zarit Burden Interview). Disease activity questionnaires (RAPID3 and BASDAI) completed by relatives assessed the patient's state. Caregiver experience questionnaires (CRA) completed by patients assessed the patient's feeling of the relative's experience.^26^

### 2.4 Data analysis

A thematic analysis was conducted by two psychologists (CD, CV), following a general inductive approach.^27^ This type of qualitative analysis enables the researcher to extract themes from the participants’ discourse in order to capture their perception of the studied subject. After reading all transcripts, the two investigators isolated themes and subthemes and created a preliminary coding schedule. After discussion with part of the research team (CD, CV, AU, EB), the coding schedule was revised.

Transcripts were loaded in the QDA‐Miner software,^28^ which was used to facilitate the analysis. First, both investigators conducted coding sessions with blinding to participant names to refine the coding schedule. After an independent analysis of patient‐relative dyad interviews, a new session involved a common analysis of participant interviews. After team discussions and modification of the schedule, the final analysis resulted in a free marginal Kappa of 0.72, which showed good interinvestigator agreement. To guarantee rigorous data, all steps of the analysis were discussed and validated by the research team, but the participants did not provide feedback on the findings.

COREQ (COnsolidated criteria for REporting Qualitative research) Checklist (File S1).

## 3 RESULTS

### 3.1 Description of the sample

Saturation was reached after 20 dyad interviews (20 patients/20 relatives): 18 partners, 1 mother, 1 friend; 13 individuals had RA and 7 SpA. Median length of disease and couple relationship were 10 (range: 1‐36) years and 28 (range: 1.5‐57) years, respectively. Other participant characteristics are presented in Table 2. Information on each dyad is in Table 3.

**Table 2:** Characteristics of patients and relatives

	Patients (N = 20)		Relatives (N = 20)	
	N	Median (range)	N	Median (range)
Age		63.0 (27‐79)		60.0 (39‐83)
Sex (women)	13		8	
Education				
High school or less	12		13	
Attended college	8		7	
Occupational status	3		7	
Employed	3		7	
Retired	13		11	
Disabled or unemployed	4		2	
At least one child	16		17	
RAPID3^a^ [0‐30]		9.3 (2.0‐18.7)		14.3 (5.5‐18.3)
BASDAI^b^ [0‐10]		5.6 (1.3‐7.0)		7.0 (2.2‐8.0)
Comorbidities^c^ [0‐18]		1.5 (0.0‐6.0)		‐
HADS^d^				
Anxiety [0‐21]		5.5 (0.0‐19.0)		8.0 (3.0‐17.0)
Depression [0‐21]		5.0 (0.0‐11.0)		5.5 (0.0‐13.0)
CRA^e^				
Self‐esteem [0‐4]		3.1 (2.3‐3.7)		3.0 (1.4‐4.0)
Financial impact [0‐4]		1.2 (0.0‐4.0)		1.0 (0.0‐3.0)
Time impact [0‐4]		1.4 (0.0‐3.2)		1.1 (0.0‐2.8)
No family support [0‐4]		1.9 (0.4‐3.0)		1.4 (0.4‐2.6)
Health impact [0‐4]		1.0 (0.0‐2.3)		0.9 (0.0‐3.0)
Zarit [0‐88]		‐		13.5 (0.0‐40)

a Routine assessment of patient index data.

b Bath Ankylosing Spondylitis Disease Activity Index.

c Groll index.

d Hospital Anxiety Depression Scale.

e Caregiver Reaction Assessment. RAPID3, 0 to 30 (high activity); BASDAI, 0 to 10 (high activity), HADS, 0 to 21 (high level of anxiety or depression), CRA self‐esteem dimension, 0 to 4 (high level self‐esteem); CRA other dimensions, 0 to 4 (high level of negative impact); Zarit, 0 to 88 (greater burden).

**Table 3:** Description of each dyad

Dyad	Patient's sax	Patient's age	Disease	Year of diagnosis	Relative	Relative's sex	Relative's age	Length of relationship
2	Man	64	Spondyloarthritis	2000	Spouse	Women	60	5
3	Woman	66	Rheumatoid arthritis	2005	Spouse	Men	64	43
4	Man	62	Rheumatoid arthritis	2010	Spouse	Women	58	38
5	Woman	78	Rheumatoid arthritis		Spouse	Men	83	54
6	Man	65	Rheumatoid arthritis	2000	Spouse	Women	60	43
7	Woman	41	Spondyloarthritis	2014	Spouse	Men	46	22
8	Man	73	Rheumatoid arthritis	2008	Spouse	Women	77	50
9	Woman	37	Spondyloarthritis	2005	Spouse	Men	39	18
10	Woman	67	Rheumatoid arthritis	1976	Spouse	Men	69	8
11	Woman	79	Rheumatoid arthritis	1990	Spouse	Men	79	57
12	Woman	58	Rheumatoid arthritis	2004	Spouse	Men	58	35
13	Man	44	Spondyloarthritis	2005	Friend	Women	39	4
14	Man	60	Rheumatoid arthritis	2008	Spouse	Women	59	37
15	Woman	34	Spondyloarthritis	2011	Spouse	Men	42	12
16	Woman	60	Rheumatoid arthritis	2012	Spouse	Men	52	3
17	Woman	78	Rheumatoid arthritis	1988	Spouse	Men	77	45
18	Woman	62	Rheumatoid arthritis	2009	Spouse	Men	66	41
19	Woman	27	Spondyloarthritis	2008	Daughter	Women	50	30
20	Woman	66	Rheumatoid arthritis	1979	Spouse	Men	66	17
21	Man	68	Spondyloarthritis	2009	Spouse	Women	67	20

### 3.2 Dyad interviews

The analysis revealed 4 main themes: the disease ‘lived’ together (new role of the relative, communication around the disease within the dyad), impact of the disease on the relationship, social impact of the disease on the dyad and shared difficulties (social isolation, difficulties in having plans, worries for the future), and difficulties and needs of the relative.

#### 3.2.1 Disease lived together

In the present sample, dyads coped with the disease together and described the relationship of partners as complementary and knowing.

##### New helping role of the relative

To live the disease together as a ‘working team’, the partners (n = 19) explained the new helping roles of relatives. The most common role of the relative was to provide help in physical tasks, especially in activities of daily living (n = 10).

- *‘When he sees I am going to open the bottle, he says to me: “give it [the bottle] to me.”’ (Patient [P10])*
- *‘So that is, he is going to help me in managing the home, also, for laundry…’ (P9)*

Understanding and mutual support, preventing the patient from the feeling of being a burden was also mentioned (n = 2).

- *‘The only strength we have, is to have a relative, to whom we can ask without having the feeling of being a burden for everybody.’ (P13)*
- *‘In the morning, the first one who gets up waits for the other. We support each other.’ (Relative [R6])*

Relatives were sometimes (n = 5) described as positive and acting as a driving force. They then became ‘family responsible’.

- *‘She was also there, I would say to boost me a little bit when I am down, with breakdown…’ (P13)*
- *‘I say to him “never mind.” I have another perspective about the disease than him. I always try to be positive. I say to myself, you should live from one day to the next and take life as it comes. We live as we can, we live it up.’ (R4)*

Relatives also could have numerous roles in medical care. They could take part in medical decisions, be present during consultations, help in adherence to treatment, help in searching for information, provide or search for some assistance for treatment administration or be a counsellor (n = 19).

- *‘It's me who does the research on the internet, who gets to the hospital.’ (R9)*
- *‘She helps me do the injection; I prepare the injection, she gives it [the injection] to me, checks whether liquid is good…not altered. She gives me the alcohol, the pad…’(P21)*

Relatives highlighted that support and help was normal (n = 2), and one relative thought that the term caregiver was not appropriate and that a caregiver was a foreigner not close to the family.

- *‘What bothers me is, he is afraid to bother me.’ (R2)*
- *‘In “caregiver,” I find this is too much, because, as a spouse, I found this is normal. “Caregiver,” I’ve got the feeling that this is someone foreign…’ (R18)*

##### Communication around the disease

Dyads explained reasons to talk or not about the disease. In total, 14 dyads explained that they usually do not talk about the disease. The first reason mentioned was to try to forget it and not to focus on it (n = 9).

- *‘What I try to have as an attitude is to…not to talk too much about it [disease], to not focus on it…I have got the feeling that talking would amplify it [disease].’ (R12)*
- *‘No, but I do whatever I can to forget it [disease], in everyday life. There's no use thinking about it, whining or whatever.’ (P6)*

They try to affirm that life was not only the disease (n = 5).

- *‘It [disease], does not take a central place in our life.’ (R20)*
- *‘Life is not on the disease…I do not spend my life thinking of that.’ (P18)*

Sometimes they preferred not to talk of something that is difficult to accept (n = 1).

- *‘We try to withhold a little bit, because, he does not really accept it [disease], I think*.’ *(R4)*

Not talking also respects the need of the patient to be alone (n = 3).

- *‘When he has a lot of pain, he needs to be alone a little bit. I know him, so…I leave him in his corner.’ (R8)*

Not annoying others is also an important reason (n = 4).

- *‘We do not talk of that [disease]. That's it…I do not want to annoy him either.’ (P18)*
- *‘I had the feeling of annoying you. I said “anyway, I have nothing to say,” because besides talking of the disease, I have nothing else to say.’ (P13)*

With time, words are not always needed (n = 1).

- *‘Not anymore, now. At the beginning, yes.’ (P6)*
- *‘Since he has been sick, I have learned to know the disease.’ (R6)*
- *‘Sometimes, we do not need to communicate with a lot of words. I see he is not well.’ (R6)*

However, for other dyads or at other times (n = 18), talking about the disease is important in daily life to improve knowledge about the disease together or for security reasons.

- *‘It [*talking about the disease] *is absolutely not taboo…We talk of it easily. Whether it is on a daily basis… say …, pain is here, how much, or where…’ (P19)*
- *‘I have my nurse who cares for me and says to me: “take care, get up…”’ (P21)*
- *‘Yes, we talk of it [disease] a lot…it's me who searched on the internet.’ (P9)*
- *‘I say everything, because I say to myself, if ever anything should happen, he will know.’ (P7)*

A patient also highlighted how asking for help was difficult.

- *‘To apologize all the time, not to dare asking…to learn to accept to ask things, yes, I think this is that also.’ (P13)*

#### 3.2.2 Impact of the disease on the relationship

Most dyads (n = 16) did not feel the IA had changed the relationship.

- *‘It has changed our way of living, yes, definitively. But not our relationship.’ (R4)*

For some, the disease had even strengthened the relationship (n = 2).

- *‘For us, we have become closer, anyhow.’ (P13)*
- *‘We have become even more close. We try to spend more time together, to do more things together than before. We know we are closer than we were before.’ (P7)*
- *‘There is gratitude with respect to the couple’ (R7)*

However, if they were positive regarding the dyad relationship, they also were aware and acknowledged some tensions because of the disease (n = 14). Nine dyads considered that the disease received too much attention in the relationship.

- *‘And then a feeling of invasiveness, I think of the disease.’ (R13)*
- *‘Anyway, I have nothing to say, because besides talking of the disease, I have nothing else to say.’ (P13)*

Lack of communication sometimes created tensions and misunderstanding (eg one patient explained how she ‘locked herself up’, felt tense and finally guilty, while her husband recognized he did not always understand what happened; n = 11).

- *‘One should really insist on making her say “yes, that's right, I am not well.” I do not realize…when we don't do some things and I don't understand why. I have difficulties understanding. I don't ask too much either, I could look for, insist.’ (R15)*
- *‘Because I don't say it [I am not well], I think I lock myself…that is, I am a clam. As I do that, I feel tense… and I could not bear anything and … this makes him nervous because he sees I am locked…. I blame myself and then when he is back, even he has not said anything, I am on the defensive.’ (P15)*

#### 3.2.3 Difficulties and needs of the relative

Difficulties and needs of the relatives were rarely raised during dyad interviews. Knowledge of the disease and the patient's symptoms were an important need expressed by relatives (n = 10).

- *‘Since he is sick, if you want, I have learned to know the disease. I have been forced to, if you want? to know everything to understand him, also.’ (R6)*
- *‘I want to be informed as much as she is on the disease. I know his pain, I know his pain locations, I know his flares, I know…’ (R7)*
- *‘We assume that if I forget some things, he can think of them [things] and conversely. So, each of us can ask questions.’ (P7)*

Sometimes the disease and its consequences were not well accepted by relatives (n = 5).

- *‘Sometimes, I am tired anyway and he doesn't really want to acknowledge it.’ (P5)*
- *‘I had someone dynamic, sparkling, but some days, she is at “2 miles an hour.” I say to myself, wait, it is not helpful to run, she won't follow.’ (R16)*

Relatives mentioned that not being able to help was frustrating and finding the right way to help and the right balance to provide help or not was not always easy (n = 5).

- *‘We face some depressive situations, not being able to help, not having the right gestures, not being able to have the right attitudes. It is quite difficult to live that with a relative.’ (R16)*

Relatives were conscious that they should not help too much (n = 3)*.*

- *‘I am sure I prevent him to… I am conscious that sometimes I am too* supportive, I intervene too much*.... Sometimes, I see her, and I still let her… I am aware that, first she won't like that because it brings to light she can't manage it… this is classic.’ (R18)*
- *‘I also don't want to let him doing everything. I don't want that he has this burden.’ (P18)*

Worries of relatives were also mentioned *(*n* = 5).*

- *‘We don't have peace of mind. When she is alone at home. If she falls, she can't get up. It is a problem. It worries me.’ (R11)*
- *‘I felt responsible for everything that happens.’ (R3)*
- *‘I was not conscious, some people…I understood they were close, but I didn't understand they could worry for me.’ (P13)*

#### 3.2.4 Social impact of the disease on the dyad and shared difficulties

##### Social isolation

Eight dyads expressed the feeling of social isolation. Going out or entertaining at home was difficult because of the disease symptoms. Because dyads cancelled several invitations or did not often go out, they felt they were forgotten.

- *‘There are not a lot of people at home because he goes to bed very early.’ (R6)*
- *‘Yes, when you have cancelled 3 times…one doesn't invite you anymore because otherwise you‘ll cancel again. But if I cancel, this is not because I don't want it [to go out]; it's because at that particular time, I couldn't.’ (P19)*
- *‘They have left us a little bit, our friends. Because, we can't often go out. Sick individuals, they are shut a little bit out.’ (R17)*

Sometimes this isolation was because patients needed to be alone (n = 3).

- *‘When he is in pain, he needs to be isolated a little bit.’ (R6)*

Invisibility of the disease could also create misunderstandings and be a reason to feel socially isolated (n = 9).

- *‘Which is difficult is that, this is a disease, you don't see. And thus, the other one's [stare] can be really hurtful.’ (R19)*
- *‘People don't see you as sick. They see you as a deadbeat.’ (P7)*

People around did not really understand the dyad difficulties because they do not know the disease or do not realize what it means to live with it (n = 4).

- *‘I think there are those who don't want to understand even when they know and those who are not conscious of the magnitude that takes in daily life.’ (R6)*
- *‘As long as you are not sick, your joints function well, you don't realize.’ (P12)*

##### Difficulties in having plans

The unpredictability of the symptoms made it difficult to organize daily life, and plans had to be modified or cancelled (n = 9).

- *‘Because, we say to ourselves, if we make a reservation somewhere, will she be fine? at that moment, she is quite well but there are moments when she is not.’ (R3)*
- *‘It is almost, we live from day to the next. We can't have long term plans anymore. Traveling — this is almost finished.’ (R6)*

##### Worries for the future

A total of 14 dyads expressed concerns about future (eg in early disease, at the beginning of the health care or towards patients work).

- *‘We worried though, because we didn't know at the beginning, we were afraid.’ (R14)*
- *‘I would be annoyed to be unemployed. This is part of my concerns. Today, I am doing pretty well, I have a shelter, I have all that. Do I risk tomorrow, because I can't find a job because I won't be able to do it [job]?’ (P16)*

Dyads of patients with RA and AS described the consequences of the disease differently, but the number of occurrences of the other themes of their experience and communication about their life with IA did not differ.

For example, irritability and depressed mood was more frequently described by dyads of patients with RA (100%) than SPA (71.4%), *P* = .04. Dyads of patients with RA (76.9%) recall the past before the disease with regret more frequently than SPA (28.6%), *P* = 0,036. However, dyads of patients with RA (23.1%) evoke a lack of control and management difficulties in relation to the disease less frequently than dyads of patients with SPA (71.4%), *P* = .036.

## 4 DISCUSSION

The current qualitative study offers new insights into understanding the perception of patients and relatives of their shared life with IA disease and their new roles and interactions and is a first step to develop interventions to support them.

### 4.1 To live the disease together as a ‘working team’—dyads explained the new roles of the relative

New helping roles of the relative described agree with the domains of illness work or activities that are relevant for managing lifelong diseases in the Vassilev et al study.^29^ Indeed, they differentiated everyday work (household activities or occupational labour), emotional work (reassurance and companionship) and illness‐specific work (medical care).

The relative often provides help for gestures or activities (eg opening a bottle, driving). Handling household tasks not previously assumed therefore changes the roles inside the family. In the Matheson et al study, many partners living with an individual with RA felt that traditional sex roles had ‘reversed’.^10^ Conversely, sometimes individuals with RA said they did not alter their roles, because of a bad conscience or because their sexual identity was threatened.^30^

The relative can endorse a role of emotional support. He/she can be supportive, understanding and act as a driving force while preventing the patient from feeling that they are a burden. Preventing the feeling of not being a burden despite the impact of the disease on the dyad's everyday life was important for many participants. Protecting the relative's feelings was also important to the patient. In one dyad, the patient did not want to talk about her disease for fear of annoying her relative, and the relative was annoyed because she was afraid to bother him (dyad 2). Emotional support is less visible than other assistance tasks but is of primary importance in IA. In long‐term conditions in general, emotional support helps patients not to feel cared for and maintain their perception of independence and identity.^31^ Several surveys have highlighted the impact of the emotional support of the partner on psychological state. In individuals with arthritis, the presence of a partner has a direct, favourable effect on psychological functioning.^32^ Increased satisfaction with the spouse reduces the likelihood of feeling helpless in dealing with daily pain.^33^ Similarly, a supportive relationship seems to encourage the adoption of better coping strategies, which leads to better psychological adjustment.^34^

Relatives can have numerous roles in medical care, and thus, they really live the disease together with the patient. Relatives are often positive and are a source of motivation for the patient to do activities and take care of themselves. This theme was also highlighted during the development of a measure of dyadic efficacy for married women with RA and their spouses. Several items address this theme: ‘maintain positive attitudes’, ‘keep each other's spirits high’ and ‘focus together on the good things in your life’.^35^ These different themes were all addressed by our dyads during interviews. Relatives can also help in the search for information. We did not especially investigate the distribution of the search for information within the dyad, and when this topic emerged, it was only to mention that the relative was responsible for managing the information. However, Mazanderani et al showed that when one member of a couple (eg the patient) avoided or ignored information, the other usually compensated by taking on the responsibility of managing information. Therefore, understanding the disease is viewed as a shared rather than individual concern.^17^

The last idea of the roles of relatives is that for relatives, support and help is something normal. They found the term ‘caregiver*’* inappropriate *(‘this is too much’)* and minimized their role, comparing it to caregivers of more dependent patients. This was also the case for Knowles et al The relative role of patients living with a long‐term condition was described in terms of familial responsibilities but not ‘caring work’,^31^ and relatives had difficulties identifying themselves as carers.^31^ In another study, some family members of patients with multiple sclerosis explained that their caring function was included in their relationship with the patient. They also reported fears of threatening the identity of the patient when they were designated as carers.^31^

### 4.2 Communication around the disease

#### 4.2.1 Dyads explained reasons to speak or not of the disease

The first reason to avoid talking about the disease is to try to forget it and not focus on it. Partners tried to affirm that life is not only the disease. This coping strategy can be used in interventions. For example, one of the positive results of an emotion‐focused group intervention for patients with rheumatic disease was that in focusing on topics related to life rather than disease, patients had the feeling of being recognized as more than the disease.^36^

From patients’ perspectives, the willingness to avoid disturbing others is an important reason for not talking about the disease. Beyond the willingness to avoid talking about the disease, patients often struggle to hide difficulties and fulfil their own and other's expectations.^36^ They also feel guilty if they have to say no and try to avoid such situations or push themselves too far.^37^ Patients who recognized the importance of talking to someone about the disease prefer to talk to other people with arthritis or health professionals because they do not want to bother family or friends.^37^

However, not talking about the disease should also be agreed upon and understood because the absence of communication can create tensions and misunderstanding.

Quality of communication has been associated with better quality of life in studies involving patients with rheumatic diseases. The mutual engagement of partners in conversations through responses characterized by empathy, authenticity, validation and empowerment was found to predict a lower level of depression and anxiety as well as physical disability and affected arthritis.^38^, ^39^

### 4.3 Impact of the disease on the relationship

Some dyads mentioned that the disease strengthened the relationship, which agreed with one study examining the positive effects of illness on relationships among patients with RA,^40^ but in our study, nine dyads considered that the disease had too much attention in their relationship. These dyads also acknowledged some tensions, especially because of difficulties in communicating about the impact of the disease.

Lack of agreement on the consequences of the disease is often not negligible in couples including a person with RA.^5^ Both over‐ and underestimations of the patient's functional disabilities by the spouse were found associated with the patient's poorer mental health status 6; conversely, couples’ congruence concerning women's control over RA consequences predicted better psychological adjustment.^7^ Fatigue is particularly difficult to manage in the couple relationship.^5^ The patient's lack of expressivity can cause misunderstanding but also a feeling of lack of trust. This was not mentioned in the present study, but patients’ perceived inability to meet spousal expectations contributes to depressive symptoms^41^ and has an impact on the relationship.

### 4.4 Difficulties of the relative

A good knowledge of the disease is an important need expressed by relatives. As in the Matheson et al study, partners wanted a joint approach to treatment involving and recognizing the partner and focusing on the couple rather than just the patient.^10^ One relative explained that he was responsible for searching the Internet.

Difficulties of relatives were seldom raised by dyads. However, difficulties in providing help were mentioned: first, because relatives felt helpless when they were unable to support the patient; and second, because finding the right way to help and the right balance to provide help or not is not always easy. For example, spousal support can increase depressive symptoms in patients expressing a high importance of completing activities independently.^42^

Relatives also can be frustrated with not being solicited. To assume a role of support is also part of the relative's identity and responsibility, and this needs to be valued and recognized inside the dyad. Taking care of others can also have emotional, physical and social benefits, leading to increased happiness and increased sense of social connectedness.^43^

### 4.5 Social impact of the disease on the dyad and shared difficulties

Social isolation is an important concern. One dyad described the social impact of the disease as ‘*a restricted life*’. Partners reported they had given up recreational shared activities and had difficulty making future plans.^10^

The study has some limitations. First, the sample was not representative of all dyads of individuals with IA in that our dyads were motivated to participate because relatives were already involved in the management of the patient's disease. However, they acknowledged some tensions, and the interviews informed on the coping strategies they used. The mean age of the sample was high (only six patients and nine relatives [20 patients/20 relatives total] were under 60 years old), which can also affect the results. Second, difficulties of relatives were not much developed by the dyads perhaps because relatives were invited by the patients and they put aside their problems. Finally, we do not have any data on refusal to participate for the above reasons and because interviewing a dyad about their disease experience can be difficult for them and rheumatologists approached only couples or patients they knew and only when they believed this was appropriate. Furthermore, they never insisted when they felt the patient and the relative were not motivated.

However, one originality of the study is that dyads have rarely been interviewed together and they had the opportunity to explore themes, express their difficulties or mistakes or how they cope together.

### 4.6 Implications for interventions

Dyads provided insight into the coping strategies they used. These findings can inform health‐care professionals regarding the provision of care for couples who are coping with IA and can have implications for TPE (Table 4):

- Recognizing the role of the relative when medical decisions are shared with professionals
- Highlighting the importance of teamwork in managing the disease
- Providing knowledge of the disease to relatives: information about arthritis symptoms such as fatigue, pain, low mood, anger, disability, invisibility of symptoms and unpredictability of the disease because they are subjective and sometimes difficult to understand by others
- Recommending discussions about relative roles, willingness to live the disease as a team and positive aspects of support but keeping in mind the ambivalence of the feelings of guilt or gratitude of the patients and guilt of the relative
- Addressing reasons to avoid speaking about the disease mentioned by dyads and encourage focusing on topics related to life rather than disease because life is not only the disease
- Working on patient communication; helping them express emotions; making them aware of their own needs, taking care of themselves and their health; working on how to express their needs to other people; learning to say no and to release feelings of guilt; and revising the requirement to fulfil too‐high expectations
- Supporting relatives to improve communication skills (eg asking whether the patient is sure they do not need help instead of deciding for them), respecting the patient's need to be alone or be a source of motivation
- Improve cognitive‐behavioural skills of both patients and relatives (eg coping strategies such as distraction, positive outlook and restraining negative emotional reactions and planning enjoyable activities)
- Trying to find strategies to increase social participation and recreational activities or to deal together with the unpredictable nature of arthritis.

**Table 4:** Suggestions for interventions according to themes addressed by the dyads

	Targets for interventions		
	General	Communication	Knowledge of disease
Themes addressed by dyads *To ‘live’ the disease together as a ‘working team’*	Highlight the importance of team work in managing the disease Improve cognitive‐behavioural skills of both patients and relatives (eg coping strategies such as distraction, positive outlook and restraining negative emotional reactions, and planning enjoyable activities)	Discuss the changes in the roles of relatives and patients Discuss the different types of support needed and provided by the relative	
*Impact of the disease on the relationship*		Discuss reasons to avoid speaking about the disease and that lack of expressivity can be felt as a lack of trust by the relative	Communicate on the impact of the disease to improve the couple's congruence on perception of symptoms and control over IA
*Difficulties of the relative* *Find the right balance to provide help or not*	Favour a joint approach to treatment involving and recognizing the partner, and focusing on the couple	Discuss the positive role of support and difficulty in asking	A good knowledge of the disease is an important need
	**Behaviour or cognitive advice**	**Relatives’ skills**	**Patients’ skill**
*Communication around the disease inside the dyad*	Do not focus on disease (life is not only the disease) Try to be optimistic and to accept	Support relatives to improve communication skills: Ask whether the patient is sure they do not need help instead of deciding for them; Respect the need to be alone Empathy, authenticity, validation	Help patients express emotions; make them aware of their own needs, taking care of themselves and their health; work on how to assert their needs to other people, learn to say no and to release feeling of guilt and revise the requirement to fulfil too‐high expectations.
*Social impact of the disease on the dyad and shared difficulties*	Try to find strategies to increase social participation and recreational activities		

## 5 CONCLUSION

This study highlighted the importance of recognizing the role of the relative in the management of IA disease, especially when medical decisions are shared with professionals. A joint approach to treatment is a basis for coping with the disease. This supposes (a) discussions about relatives’ new roles to delimitate and clarify them, (b) patients’ communication skills (eg ability to express emotions and needs and to say no) and relatives’ communication skills (eg asking whether the patient is sure they do not need help, respecting the patient's need to be alone or be a source of motivation) and (c) a good understanding of each other, which can be improved by providing information on the disease and coping strategies for both the patient and the relative.

## CONFLICT OF INTEREST

Financial support from *Roche Foundation (unrestricted grant)*.

## Data Availability

Data are available on request from the authors.
