# Supplementary material for: Patients and relatives coping with inflammatory arthritis: Care teamwork
Source: Health Expect. 2019 Nov 27;23(1):137–47. doi: 10.1111/hex.12982 (PMC6978873; doi:10.1111/hex.12982)
Supplement: Supplementary file 1 [file HEX-23-137-s001.docx]

Supplementary file 1
